# A Femininomenon: Leadership Development Through Representation On‐Screen

**DOI:** 10.1002/yd.20658

**Published:** 2025-02-25

**Authors:** Kathleen Callahan

**Affiliations:** ^1^ Department of Leadership and American Studies Christopher Newport University Newport News Virginia USA

## Abstract

Historically, films and television centered men, but there has recently been a shift toward focusing on women and people of color (and women of color) in leading roles. Films and shows like *Black Panther*, *Barbie*, and *Ashoka* reflect this trend, offering more complex stories and diverse representation. Despite progress, stereotypical gender roles persist in many portrayals, but contemporary films and television shows increasingly challenge these norms. The shift toward intersectional narratives in pop culture encourages broader views of leadership and identity. When popular culture predominantly highlights the stories of (White) men, it perpetuates the belief that leadership is exclusive to them. Addressing the complex challenges of the 21st century and beyond requires a more diverse range of stories that also deserve recognition and inclusion. Leadership educators and practitioners can enhance learning by encouraging observation and using relevant pop culture examples to bridge leadership concepts and theory with lived experiences.

## 1 Introduction

Xena and Buffy were among my favorite characters on television growing up, and I wasn't the only one. These were characters young girls in the millennial, and GenX generations were drawn to as strong female leads, some of the first we saw in protagonist roles and unlike the many roles held by women in the past. However, in the United States, much of pop culture seen on screen in the past includes a hero's journey as seen through the eyes of men. The many family sitcoms and prime‐time dramas with men at the center were the norm. However, in recent years, we have begun to see a narrative shift more towards women and people of color on screen in protagonist roles.

Haber‐Curran and Tillapaugh (2017) asserted, “…there has continued to be a great deal of connection between leadership and those having power and authority through positional leadership roles, which historically have been White men,” (p. 16). They attempted to broaden our awareness and motivate a shift toward an intersectional, nuanced, and complex view of identity and leadership (Haber‐Curran and Tillapaugh 2017). In 2022 and 2023, audiences flocked to see films like *Woman King*, *Black Panther*, *Everything Everywhere All at Once*, and *Barbie*, and to see musicians like Taylor Swift and Beyonce. In 2024, in the United States, women dominated the music charts, Kamala Harris was nominated as a presidential candidate, and women won a majority of Olympic medals (Treisman 2024).

Younger generations look up to these women and see their own potential in these intersectional narratives on their screens. We see intersectional representation in many forms of popular culture, such as music, film, television, social media, sports, and beyond. This article will emphasize and highlight leader identity beyond what we have known to be the dominant male‐centric journey on screen, explicitly the shift of “the journey” concerning women identifying characters, their intersectionality, and underrepresented identities in leadership.

The traditional superhero films and early *Star Wars* films have shifted towards focusing on women and people of color in those same fictional universes. The hero's journey on screen has run dominant in the past, as men have held a majority of the protagonist and main speaking roles on screen (Lauzen 2024). These stories are plentiful in film, and protagonists like Luke Skywalker and Harry Potter consist of boys/men going through great trials and tribulations to become heroes (Campbell 1990). These are stories shared early in life that inform implicit ideas of what it looks like to be a leader.

However, women in the past have rarely been seen in these stories beyond a supporting character, if at all. Stories of women are starting to be more visible in mainstream pop culture. Women directors and producers offer more complex storylines for women and people of color, such as *Barbie*, *Black Panther*, and expanding the *Star Wars* universe with women like Ahsoka Tano and Rey, or on television and streaming like *Abbott Elementary* and *We Are Lady Parts*. These characters provide women with storylines that show women as protagonists with complex journeys. These are not the first women characters to dominate pop culture, but the diversity and complexity of the women taking the lead reflects a desire of the audience to see women on their own journeys.

Women's stories and representation in fictional “universes” have also seen a slight shift over time in which films have progressively moved from more homogenous communities, as seen in Tolkien's *Lord of the Rings* and early *Star Wars*, to more diverse worlds within Marvel's *X‐Men* and television series such as *Star Trek*, *Bridgerton*, and *Heartstopper*. Over time, writers have begun setting their narratives in real‐world settings that include the human experience, and the many intersecting identities characters hold. This inclusion in mainstream pop culture encourages viewers to see the world with the same openness and possibility.

At the same time, we see women in more complex ways than in the past, when women were featured as either “the bitch” or “the ditz” (Fortini 2008). Unfortunately, even in these examples, we still see stereotypical gender roles situated in a patriarchal system, but with a critical lens, there are lessons that can be extrapolated with the help of knowledgeable educators and practitioners. Even in 2023's box office hit, *Barbie*, a film that seemingly empowered women around the world to see themselves as Barbie by offering Barbieland as diverse and inclusive, it is the stereotypical White Barbie who navigates the patriarchy with certain privileges (Chatterjee and Pitre 2023).

## 2 Overview

Marturano et al. (2013) claimed, “…leadership is a collaborative, relational activity, and (we) believe that the study of leadership must likewise be a dialog, especially a dialog across lines often perceived to be rigid and impermeable boundaries,” (p. 2). One way to do this is through socially just leadership education, training, and critical conversations. Leadership is interdisciplinary and human, and we can observe it both directly and indirectly through film and television. Film and television shows are often used in leadership education, development, and training. Leadership learners can connect with characters from their favorite movies and shows in a way they may not be able to connect with leadership concepts from a textbook or journal article.

Long (2017) discussed the benefits of using films as case studies to teach leadership concepts and encouraged critical conversations around leadership, explaining, “More than simply containing stories about leadership, films contribute to particular leadership discourses by defining leaders, followers, the relationship between them (complete with power asymmetry), and the purpose of leadership,” (p. 75). Adopting this orientation furthers leadership learners' ideas about, experiences of, and how they might view and practice leadership and followership in their own lives. The same could be said about television shows, especially with a series running over several years establishing some of the characters that grow alongside the viewer. The ability to see a favorite character grow over time, through adversity and crisis, can help the viewer better understand and relate to others' experiences.

Jenkins (2012) explored instructional strategies and pedagogy including film, to help students gain self‐awareness and develop as leaders in the classroom space. Callahan et al. (2007) discussed the use of different mediums of pop culture, such as film and television, as ways, to help leadership learners make meaningful connections to characters as they think and reflect on the leadership process. Additionally, authors such as Scott and Weeks (2016) shed light on how they utilize film to teach specific concepts like authentic leadership. There is plenty of research on the effectiveness of the use of media in the classroom.

However, for leadership learners, especially those of generations “Z” and “Alpha,” this use of technology might become a useful pedagogical tool due to the digital literacy of these generations. In this article, the term *leadership learner* is used rather than student intentionally. Everyone watching films and television shows is influenced by what they observe, and many are learning leadership skills and lessons simply through exposure (Guthrie and Jenkins 2018). Educators and practitioners may not be bound to a university but have the influence to teach, train, and develop individuals outside of the classroom or the university. Leadership learning happens everywhere. I encourage educators and practitioners to find examples to fit their local context.

Leadership involves being in a relationship with one another, is accessible to everyone, and encourages people to view themselves as potential leaders in their everyday actions and interactions (Komives et al. 2005). The focus is on developing leadership capacities through experience, reflection, and engagement with others. Roberts (2007) called us to take, “…small steps that take us out of our routine ways of seeing the world, exposing us to new possibilities and bringing us to a depth of perspective that is more authentic, believable, and trustworthy,” (p. 3) through conviction. Leadership educators and practitioners can design courses, trainings, and programs for leadership learners. They can capitalize on what these learners already know: pop culture. Using film, television, and streaming shows, we can meet learners both where they are in their developmental journey and physically, in front of a screen. We can then serve as a resource as they develop their leader identity through coursework, experience, and practice. Educators who believe, “Leader identity is considered to be both a *precursor* and an *outcome* of leader development” (Johnson et al. 2023, 23) may have a corresponding responsibility to consider how we can use media like film and television (which students are already familiar and comfortable with) to encourage leadership development.

Films, television, and streaming series give us a variety of genres to choose from and include both fictional and nonfictional storylines. “Dominant leadership narratives inform who can and cannot be seen—or see themselves—as a leader or engaging in leadership,” (Beatty et al. 2020, 41). Who are we regularly seeing on‐screen in protagonist and speaking roles? Lauzen (2024) and *It's a Man's (Celluloid) World* publishes an annual report containing data on the portrayals of female characters in top‐grossing US films every year since 2002. At their peak, women made up 40% of the main protagonists in 2019. Most recently, in 2023, women were only 28% of the protagonists (Lauzen 2024).

How might seeing more women's stories in film and television contribute to leadership development? How might they challenge the dominant male leadership narratives in which many leadership learners might not see themselves represented? It is already being done. With or without leadership educators/practitioners or outside influence, people are constantly being exposed to and influenced by seeing women both in fictional works and in the real world living authentically. Think about your own childhood. What TV shows and movies did you watch that impacted your life? What did you watch, and how did it influence you? Did you see people that looked like you and held similar identities? How did you see leadership and followership in action? This is a good place to start with leadership learners.

## 3 Pedagogy Using On‐Screen Examples

Leadership educators and practitioners can use diverse pedagogies to provide learners with examples and resources, helping them navigate and develop their own leadership efficacy and capacity from a “just, equitable, diverse, and inclusive” (JEDI) perspective (Guthrie et al. 2016). Leadership learners are continually learning about themselves through their consumption of pop culture. Film and television are two primary ways learners observe what they might interpret as both good and bad leadership. Additionally, they can see themselves in these different roles as seen on screen. Learners then come to educational spaces with their pop culture knowledge already “downloaded” in their minds.

What follows next includes examples and explanations of how I use this “downloaded” information and connect it to the leadership identity development (LID) model (Komives et al. 2005). Doing this can help learners understand where they are in their own development as we help connect their knowledge and application of leadership. For traditionally aged college students, their time in higher educational institutions is critical to their leader identity development; therefore, leadership educators and practitioners should be serving as resources to their students along the way.

### 3.1 Connecting Films and Television Shows to Leader Identity Development

Komives et al. (2005) sought to understand how individuals developed their leader and leadership identity. They identified adults, peers, involvements, and reflective learning as developmental influences on an individual's growth (Komives et al. 2005). Later research on the LID model encouraged a more holistic and critical examination of leadership identity (Owen 2023).

Owen (2023) clarified how understanding each of these terms is critical to leadership practitioners and educators as they work with students on their individual identity journeys. *Leader identity* is defined as, “…a sub‐component of one's identity that relates to being a leader or how one thinks of oneself as leader” (Day and Harrison 2007, 365), which Owen (2023) furthered by saying it also, “…relates to one's positionality, how it is enacted, and how others perceive and respond to it” (p. 13). How might we use film and television to help learners develop their leader identity? Some questions we may reflect upon could include the following: How might a learner see themselves or others as a leader? How do they respond to leadership? How might film or television characters help learners identify good or bad leadership and followership?

One way we can explore the LID model of development with students is by walking through each stage and including current intersectional examples in film and television. Integrating Komives et al.’s (2005) LID model with relevant leadership development theories and critical perspectives expands our understanding of how television and film characters and storylines can influence leader identity and how we might be able to use examples like those below in leadership development. As film and television characters and those behind the cameras have been diversified to include more women and people of color, we are being exposed to more complex stories than the typical hero's journey that used to dominate screens. By examining this influence, we can see how leadership learners may develop informal theories and view themselves in the stories that, in the past, only reflected dominant narratives.

Dugan (2017) framed informal theories as those that, “…reflect broader socialization systems that teach us from an early age implicitly and explicitly about how the world is ‘supposed’ to work” (p. 29). As these stories become more complex and diverse, more people can see themselves represented. As we begin to see this complexity even within ourselves and in representation through characters, we can begin to acknowledge that there is no simple story, no simple character, and no simple way to “see or do” leadership and followership.

In the LID model, Komives et al. (2005) identified the following six stages through which an individual progresses in developing a leader identity: awareness, exploration/engagement, leader identified, leadership differentiated, generativity, and integration/synthesis (see Table 1). Role models and developmental influences in the early stages of *awareness* and *exploration/engagement* stages are essential for individuals to begin seeing themselves as potential leaders or simply developing their own identities. Bandura's (1977) social learning theory explains how individuals learn behaviors by observing and imitating others, underscoring the significance of media portrayals. For women and people of color, who may, in the past, have fewer real‐world leadership role models due to systemic barriers and exclusion, television and film characters offer accessible examples.

**TABLE 1:** Leadership identity development (LID) stages and connections.

Stages of LID model	Brief overview	Leadership theory/Concept connection	TV/Film connections
Awareness	Recognizing/Exposure to leadership	Social learning theory (Bandura 1977); critical race theory (Delgado and Stefancic 2017); intersectionality (Crenshaw 1989)	Olivia Pope in *Scandal* (Hulu) Kamala Khan in *Ms. Marvel* (Disney+)
Exploration/Engagement	Intentional involvement, experience, taking on responsibility, identity development		
Leader identified	Trying new roles, individual responsibility, identifying skills needed, leadership as positional	Transformational leadership (Bass 1985); feminist leadership theory (Sinclair 2014)	Jessica Pearson in *Suits* (Netflix)
Leadership differentiated	Seeing leadership as more complex; team‐oriented, belief in nonpositional leadership, commitment to community	Adaptive leadership (Heifetz 1994); feminist leadership (Eagly and Carli 2007); indigenous leadership models (Wilson 2008); Ubuntu (Tutu 1999)	Cristina Yang in *Grey's Anatomy* (Hulu) Nakia in *Black Panther* (Disney+)
Generativity	Active commitment to personal passion and the development to others/team, responsibility for sustainable organizations	Authentic leadership theory (Avolio and Gardner 2005); culturally relevant leadership learning (Bertrand Jones et al. 2016)	Devi Vishwakumar in *Never Have I Ever* (Netflix) Amina in *We Are Lady Parts* (Peacock)
Integration/Synthesis	Continued self‐development, lifelong learning, and striving for congruence		

*Note*: Adapted from Komives et al. (2005).

In order to investigate the complexity of such examples in pop culture, critical perspectives, and concepts, such as *critical race theory* (Delgado and Stefancic 2017) and *intersectionality* (Crenshaw 1989), can help by emphasizing the importance of representation that acknowledges the intersection of race, gender, and other identities. Similarly, in the field of leadership, the National Leadership Education Research Agenda 2020–2025 identifies centering social identities as Priority 1, encouraging researchers to move away from the dominant leadership narratives (Beatty et al. 2020, 39). Utilizing these concepts can help us explore characters like Olivia Pope in *Scandal* (Hulu) and Kamala Khan in *Ms. Marvel* (Disney+). Both provide role models to people of similar identities and challenge dominant narratives of leading by presenting leadership within the context of historically excluded racial and gendered experiences. This aligns with the idea that LID for underrepresented groups is about both adopting mainstream and Western leadership qualities and redefining leadership in ways to reflect unique experiences, challenges, and joy.

As individuals identify as leaders in the *leader‐identified* stage, the narratives and archetypes they engage with play a crucial role in shaping their leadership identity. Traditional leadership theories, such as *transformational leadership* (Bass 1985), emphasize traits like vision, charisma, and inspiration, which mainstream protagonists often embody. However, these traditional frameworks often overlook the leadership experiences of underrepresented groups. Feminist leadership theory (Sinclair 2014) critiques the dominance of Western, patriarchal leadership models and advocates for more inclusive and diverse representations. Jessica Pearson in *Suits* (Netflix) provides us with one solid example to discuss as she exemplifies a leadership archetype that defies traditional leadership models. Jessica Pearson's strategic and authoritative leadership challenges the stereotypical portrayals of Black women in positions of power, providing a more nuanced and empowering narrative.

Moving further in their leader identity development, in the *leader differentiated* stage, they begin to recognize the complexity of leadership. This stage aligns with theories of adaptive leadership (Heifetz 1994), which emphasize the need for leaders to navigate complex, adaptive challenges that require more than technical solutions. Characters like Cristina Yang in *Grey's Anatomy* (Hulu) and Nakia in *Black Panther* (Disney+) illustrate the complexities of leadership, particularly for women and people of color. Cristina Yang's leadership journey reflects the need to balance ambition with empathy and personal well‐being, which resonates with feminist leadership that advocates for leadership approaches that value relationality and care (Eagly and Carli 2007). Nakia's leadership, which is deeply rooted in her cultural identity and commitment to her people, challenges the often individualistic and Western‐centric notions of leadership, aligning with *indigenous leadership models* (Wilson 2008) that emphasize community and collective well‐being; the African concept of *Ubuntu* (Tutu 1999).

In the later stages of *generativity* and *integration/synthesis*, individuals seek to incorporate leadership into their broader sense of self and commit to it in ways that align with their values. *Authentic leadership theory* (Avolio and Gardner 2005) emphasizes the importance of self‐awareness, relational transparency, and internalized moral perspectives, which are critical for individuals in these later stages. Television series characters like Devi Vishwakumar in *Never Have I Ever* (Netflix) and Amina in *We Are Lady Parts* (Peacock) exemplify leadership that is authentic and deeply connected to their personal identities. Devi and Amina's separate journeys, though more focused on coming of age, reflect the challenges of integrating multiple aspects of identity into a cohesive leadership identity, particularly for young women of color navigating cultural and generational expectations.

*Culturally relevant leadership learning* (CRLL) emphasizes the need for leadership development frameworks that are inclusive of diverse cultural perspectives and experiences (Bertrand Jones et al. 2016). Each of the previous examples helps leadership educators and practitioners do exactly that in learning spaces. As learners develop their own leader identities, they are constantly being influenced, indirectly and directly, by pop culture, so why shouldn't educators and practitioners take advantage of mediums like film and television to build on something learners are familiar with already?

### 3.2 Learning Leadership Using Leadership Observation

It is crucial to underline the importance of providing examples that are diverse and inclusive. Devies (2022) discussed how observation allows learners to, “…evaluate power, privilege, inequity, inclusion, engagement, and other dynamics between participants in the leadership process,” (p. 102). This happens in day‐to‐day activities as well as in engagement with pop culture. Devies et al. (2024) suggested leveraging observation as a pedagogical tool for leadership learning. They delineated four quadrants, including passive and active deductive leadership observation and passive and active inductive leadership observation (Devies et al. 2024). Learners can engage in observation in these different ways to connect leadership concepts through designated film and television examples.

*Barbie* and *Ahsoka* are two excellent, on‐screen examples that can be utilized in and out of classrooms for leadership development. *Barbie*, released in 2023, dominated the summer box office, and women of all ages flocked to see the film (Gerwig 2023). Ahsoka is a character in the Star Wars universe that has only recently been explored as a character of interest. The audience has not seen many stories of women beyond Princess Leia prior to the expansion of the original six films (Filoni 2023). This expansion gives us more diversity and intersectional characters. We can look at their leadership styles, character development, and relationships with others to connect learners with leadership concepts and their own leader and leadership identity. Using assignments, in‐class discussions, programming, and/or an unstructured conversation with learners, leadership educators, and practitioners can watch films, TV shows, or clips to engage the learners in reflection using observation, as Devies et al. (2024) explained in their model.

#### 3.2.1 *Barbie*: Authentic Leadership

In this example, I connect *Barbie* using authentic leadership theory through Devies et al.’s (2024) *active‐deductive leadership observation*. One way to apply *active‐deductive leadership observation* to *Barbie* would be to provide the script of Gloria's (America Ferrera's character) speech to learners, show the clip, and then have them directly apply authentic leadership theory (Devies et al. 2024). The speech reflects the core principles of authentic leadership theory, which emphasizes self‐awareness, transparency, and a deep commitment to core values (Avolio and Gardner 2005). Authentic leaders are often characterized by their ability to connect with followers on an emotional level, fostering trust and inspiring others to act with integrity. Gloria confronts the impossible standards placed on women, addressing the contradictions and pressures they face. This speech expresses her own frustrations and realizations, which aligns with the self‐awareness component of authentic leadership (Avolio and Gardner 2005). She is deeply aware of the societal expectations imposed on her and other women, and she openly shares her struggles in a way that resonates with others.

Being vulnerable and honest about her experiences, Gloria opens a conversation with Barbie and the other characters, which helps them connect with their own feelings of inadequacy in their own context. This authenticity creates a moment of collective understanding and empowerment, as her honesty helps others see their own struggles in a new light, leaving the audience to connect in meaningful ways not just personally, but in relationship with others. Finally, Gloria challenges societal norms and expresses a desire for change, demonstrating her commitment to personal and societal values of equality and fairness. Her words encourage others to embrace their own authenticity, rejecting the unrealistic ideals imposed upon them by society, thus showing a commitment to her values and modeling the way for the other Barbies to take back Barbieland (Gerwig 2023).

*Barbie*, as an example, is also a way to view the world. In this film, Barbieland is a place where the Supreme Court is all women, the president is a woman of color, and there is no distinction between feminine and masculine jobs, roles, and abilities. We see the complex intersectionality of women in a way that no one questions gender and ability. We see women of all backgrounds, identities, and characteristics living in harmony and supporting one another. Barbieland is a world in which we don't see the harsh reality of the patriarchy, including racism, sexism, classism, and division; rather, we see people living their lives in which they seem fit to do so in an authentic way that gives them purpose. Even the Kens realize that they don't like the patriarchy as it didn't benefit them to live their lives the way they wanted (Gerwig 2023). While the film shows tremendous progression for female protagonists and inclusion of complex women, stereotypical Barbie is at the center of the film, which highlights the need for representation beyond the perception of inclusiveness.

All films, however, can be critiqued. Commentary exists in support of *Barbie* as an inclusive feminist film (Zhou 2024). Others say it is, “…a lost opportunity on what could have been a meaningful commentary on the current politics of women's rights and gender equality,” (Chatterjee and Pitre 2023, para. 19). Both explore the film in critical ways with differing viewpoints and both would serve well as additional readings for learners as they begin their own critical analysis of the film.

The Netflix documentary *Black Barbie* combats critiques of *Barbie* by highlighting the work of women of color in Mattel to bring Black Barbie to life (Davis 2023). Davis (2023) shares the story of her aunt, Beulah Mae Mitchell, who worked at Mattel and asked the CEO, Ruth Handler, why none of the Barbies looked like her. Over the next few decades, three women of color developed products that represented how girls around the world appeared beyond the original Barbie doll (Davis 2023). The analysis can serve as an additional conversation between learners and educators to establish new understandings of differing perspectives concerning representation and how a film like this can re‐activate conversations around body image, gender, skin color, ability, and the possibilities that Barbie can show the world how intersectionality works beyond the doll and into the real world.

#### 3.2.2 Ahsoka: Women in Leadership

We can also use *active‐inductive leadership observation* after teaching concepts of women in leadership and then having students apply what they learned to the character of Ahsoka from the television series *Ahsoka* and *Clone Wars* (Devies et al. 2024). For example, the topic of women in leadership focuses on the unique challenges women face in leadership positions, such as overcoming stereotypes, balancing communal and agentic traits, and navigating organizational biases (Eagly and Carli 2007). Educators would use these readings and leadership concepts as they discuss the show and Ahsoka's character, which represents many of these challenges as she navigates a male‐dominated universe made of rigid and patriarchal power structures (Filoni 2023). We've seen this universe in past films and other television shows on Disney+, but this is one of the first times we see a diverse woman character navigate it as the protagonist.

One of the barriers Ahsoka faces is the tension between strength and compassion, a duality often expected of women leaders. Research suggests that women leaders are often evaluated more harshly than men, as they must balance communal expectations (e.g., being nurturing and cooperative) with agentic traits (e.g., being assertive and competent) (Heilman 2001). Ahsoka exemplifies this balance; she is both compassionate and strong, exhibiting patience and empathy while also being a formidable warrior. This duality allows her to earn respect from her peers while maintaining the moral integrity expected of her.

Moreover, Ahsoka's decision to break away from the Jedi Order reflects the “double bind” many women leaders face when challenging established norms (Catalyst 2020; Filoni 2023). Women who deviate from traditional expectations often face scrutiny or backlash, which mirrors Ahsoka's own experience of being rejected by the Jedi Council (Filoni 2023). Her resilience in the face of institutional failure highlights a leadership style that is both adaptive and resistant to the status quo, qualities that women in leadership often need to thrive in environments that may not initially support them (Catalyst 2020).

## 4 Recommendations, Critiques, and Implications

Using pedagogical tools like leadership observation can help educators and practitioners assist learners in reflecting on something they already know, like films and TV shows, to connect with leadership concepts. Finding and using relevant leadership models such as the LID model can assist educators and practitioners in better understanding where students are in their own development and give them tools to further their own development of their leader and leadership identity. In my experience, giving the tools directly to students, such as providing a developmental model, helps them see things in a new way. Rather than keeping it as a tool for professionals, equipping students with the knowledge will help them take ownership of their growth and development.

One constant in leadership and life is change, which means our taste in pop culture is also ever‐evolving. How often do we see educational programs based on pop culture from decades prior? Educators and practitioners have a responsibility to diversify curricular and co‐curricular resources. What sources are being used in classes, training, and development sessions? Who is represented, and do we see authors, directors, actors, and media representation from different backgrounds and experiences? As educators, we come to a space with our knowledge, values, experiences, and education.

As we educate in leadership spaces, we must continue to develop ourselves, staying up to date with new scholarship, new technologies, and teaching techniques. This means diversifying our syllabi, programs, and training to reflect our current and future students. This also means we should be teaching journal articles and texts that come from diverse perspectives rather than leadership textbooks of the late 1900s. Critical perspectives should be incorporated into how we view leadership and our pedagogy. It is just as much a responsibility of the educator to learn new trends and new ways of thinking about and teaching leadership and followership. This also implies that much of our current content may one day be out of date as well. We see plenty of “leadership examples” written about in the past that eventually turned sour when we learned they were, in fact, not great leadership examples.

Haslam et al. (2024) posited the idea of “zombie leadership” by pointing out dead ideas of leadership that still walk among us in leadership literature, practice, and education. Additionally, they clarified what should be the important points of leadership rather than the “dead ideas,” which include the following: importance of relationships and connections, followership, power through followers rather than over, and leadership as a group process and not one individual.

Finally, they provided counter strategies that include “broadening our understanding of leadership beyond leaders, to see leadership as a process to which everyone can, and needs to, contribute, to recognize that success depends upon connections between leaders and their group, and to leadership development that prioritize these objectives” (Haslam et al. 2024, 10). I argue it must stop from the top. If leadership educators and practitioners are still teaching these dead ideas, how do we expect leadership learners to critically examine leadership if we are not willing to move past zombie leadership?

***Perception isn't reality***. Does the fact that our perception of more women being represented on screen mean women are reaching equity in the film industry? Unfortunately, even with what seems to be an increase of women on screen, perception can be misleading. In the top 100 grossing films in the United States, “…the overall percentage of women in speaking roles contracted from 37% in 2022 to 35% in 2023, and the number of females in major roles remained at 38%,” (Lauzen 2024, 1). In fact, in 2019, 40% of protagonists were women, and since then, the numbers have dropped to 33% in 2022 and 28% in 2023 (Lauzen 2024). Major women characters are still younger on average than major male characters, and White women are overrepresented in 56% of the major female roles. However, Lauzen (2024) noted when women are involved in directing or writing, there is more likely to be women as protagonists and in speaking roles.

From a leadership perspective, this is another conversation to have with leadership learners. Yet we are seeing more complex and diverse women's stories on screen, and this should be noted as young people are able to see women in ways that were not available a few decades ago. Mavin (2009) noted, “How popular media represents women leaders is a powerful construction of what is acceptable and unacceptable in society,” (p. 7). Being able to see complex portrayals of women and underrepresented populations on screen shows the world these very real narratives and makes it possible for young learners to see themselves in new and evolving ways. If how we see women in roles reflects what is acceptable and unacceptable (Mavin 2009), then even when numbers don't reflect an increase in visibility, the roles, bylines, and sketches themselves are progressing with more complex stories.

***Drawbacks to fictional characters and complex issues***. The real world is more complex than the fictional one. We can oversimplify complex social issues because there is no time or space to address is fully in a fictional work. Characters cannot fully embody every experience and identity. We, as observers, must acknowledge and address this limitation as we engage in discussions. Seeking opposing or differing perspectives, like film critiques, is important to the critical analysis of any media.

***Access***. Another issue with using film and television is access. All of the platforms mentioned in this article are behind paywalls. This is a larger issue with how educators and practitioners might be able to access these resources when they are not in one place but in several. We can no longer buy physical media and store it in the library for use, as was common practice in the past (we can but to an extent). These subscriptions to services increase in price from year to year, so we must figure out how to ensure educational access and use in the future.

## 5 Conclusion

Leadership educators and practitioners should intentionally leverage film, television, and streaming media to help learners develop their leader identities, connect with leadership concepts, and critically analyze leadership and followership in various contexts. Using media with diverse protagonists, including women and people of color, enables learners to examine leadership through different lenses and challenges traditional stereotypes. By integrating contemporary pop culture into leadership development, educators and practitioners can engage learners, link theory and critical concepts to lived experiences, and broaden perspectives on leadership and followership to meet the complex challenges of the 21st century.

## References

[yd20658-bib-0001] Avolio, B. J. , and W. L. Gardner . 2005. “Authentic Leadership Development: Getting to the Root of Positive Forms of Leadership.” Leadership Quarterly 16, no. 3: 315–338. 10.1016/j.leaqua.2005.03.001.

[yd20658-bib-0002] Bandura, A. 1977. Social Learning Theory. Prentice‐Hall.

[yd20658-bib-0003] Bass, B. M. 1985. Leadership and Performance Beyond Expectations. Free Press.

[yd20658-bib-0004] Beatty, C. C. , L. Irwin , J. E. Owen , et al. 2020. “A Call for Centering Social Identities: Priority 1 of the National Leadership Education Research Agenda 2020–2025.” Journal of Leadership Studies 14, no. 3: 39–43. 10.1002/jls.21719.

[yd20658-bib-0041] Callahan, J. L. , J. K. Whitener , and J. Sandlin . 2007. “The Art of Creating Leaders: Popular Cultural Artifacts as Pathways for Development.” Advances in Developing Human Resources 9, no. 2: 146–165. 10.1177/1523422306298.

[yd20658-bib-0005] Campbell, J. 1990. The Hero's Journey. HarperCollins.

[yd20658-bib-0006] Catalyst . 2020. “The Double‐Bind Dilemma for Women in Leadership: Damned If You Do, Doomed If You Don't.” https://www.catalyst.org/research/the‐double‐bind‐dilemma‐for‐women‐in‐leadership‐damned‐if‐you‐do‐doomed‐if‐you‐dont/.

[yd20658-bib-0007] Chatterjee, B. , and A. Pitre . 2023. “Barbie: A Critical Commentary.” https://medium.com/@baishalichatterjee_49244/barbie‐a‐critical‐commentary‐50bfa1f08608.

[yd20658-bib-0008] Crenshaw, K. 1989. “Demarginalizing the Intersection of Race and Sex: A Black Feminist Critique of Antidiscrimination Doctrine, Feminist Theory and Antiracist Politics.” University of Chicago Legal Forum 1989, no. 1: 139–167.

[yd20658-bib-0042] Devies, B. (2022). “Making the World Your Classroom: Observation as a Pedagogical Tool for Leadership Learning”. In Navigating Complexities in Leadership: Moving Towards Critical Hope edited by K. L. Guthrie , and K. L. Priest , 99–108. Information Age Publishing.

[yd20658-bib-0009] Davis, L. (Director) 2023. “Black Barbie [Film].” Netflix.

[yd20658-bib-0010] Day, D. D. , and M. M. Harrison . 2007. “A Multilevel, Identity‐Based Approach to Leadership Development.” Human Resource Management Review 17, no. 4: 360–373. 10.1016/j.hrmr.2007.08.007.

[yd20658-bib-0011] Delgado, R. , and J. Stefancic . 2017. Critical Race Theory: An Introduction, 3rd ed. University Press.

[yd20658-bib-0012] Devies, B. , G. R. Mitchell , and K. Gibson . 2024. “First Come, First Observed: Utilizing Observation as a Pedagogical Tool to Transform Leadership Learning.” New Directions for Student Leadership 2024, no. 183: 91–101. 10.1002/yd.20628. 39217626

[yd20658-bib-0013] Dugan, J. P. 2017. Leadership Theory: Cultivating Critical Perspectives. Jossey‐Bass.

[yd20658-bib-0014] Eagly, A. H. , and L. L. Carli . 2007. Through the Labyrinth: The Truth About How Women Become Leaders. Harvard Business Press.

[yd20658-bib-0015] Filoni, D. (Writer) 2023. “Ahsoka [TV Series].” Disney.

[yd20658-bib-0016] Fortini, A. 2008. “The “Bitch” and the “Ditz”.” Vox Media Network. November 14. https://nymag.com/news/politics/nationalinterest/52184/.

[yd20658-bib-0017] Gerwig, G. (Director) 2023. “Barbie [Film].” Warner Bros Pictures.

[yd20658-bib-0018] edited by Guthrie, K. L. , T. Bertrand Jones , and L. Osteen , eds. 2016. Developing Culturally Relevant Leadership Learning. New Directions for Student Leadership. 10.1002/yd.20205 27870522

[yd20658-bib-0019] Guthrie, K. L. , and D. M. Jenkins . 2018. The Role of Leadership Educators: Transforming Learning. Information Age Publishing.

[yd20658-bib-0020] Haber‐Curran, P. , and D. Tillapaugh . 2017. “Gender and Student Leadership: A Critical Examination.” New Directions for Student Leadership 2017, no. 154 Summer: 11–22. 10.1002/yd.20236. 28524548

[yd20658-bib-0021] Haslam, S. A. , M. Alvesson , and S. D. Reicer . 2024. “Zombie Leadership: Dead Ideas That Still Walk Among Us.” Leadership Quarterly 35, no. 3: 101770. 10.1016/j.leaqua.2023.101770.

[yd20658-bib-0022] Heifetz, R. A. 1994. Leadership Without Easy Answers. Harvard University Press.

[yd20658-bib-0023] Heilman, M. E. 2001. “Description and Prescription: How Gender Stereotypes Prevent Women's Ascent Up the Organizational Ladder.” Journal of Social Issues 57, no. 4: 657–674. 10.1111/0022-4537.00234.

[yd20658-bib-0024] Jenkins, D. M. 2012. “Exploring Signature Pedagogies in Undergraduate Leadership Education.” Journal of Leadership Education 11, no. 1: 1–27. 10.12806/v11/i1/rf1.

[yd20658-bib-0025] Johnson, S. K. , S. E. Murphy , and R. E. Riggio . 2023. Leadership and Identity Construction: Processes, Relationships, and Outcomes. Academic Press.

[yd20658-bib-0026] Bertrand Jones, T. , K. L. Guthrie , and L. Osteen . 2016. Culturally Relevant Leadership Learning: Advancing Leadership Learning in Diverse Contexts. John Wiley & Sons.

[yd20658-bib-0028] Komives, S. R. , J. E. Owen , S. D. Longerbeam , F. C. Mainella , and L. Osteen . 2005. “Developing a Leadership Identity: A Grounded Theory.” Journal of College Student Development 46, no. 6: 593–611. 10.1353/csd.2005.0061.

[yd20658-bib-0029] Lauzen, M. M. 2024. “It's a Man's (Celluloid) World: Portrayals of Female Characters in the Top Grossing Films of 2023.” https://womenintvfilm.sdsu.edu/wp‐content/uploads/2024/02/2023‐its‐a‐mans‐celluloid‐world‐report.pdf.

[yd20658-bib-0030] Long, B. S. 2017. “Film Narratives and Lessons in Leadership: Insights From the Film & Leadership Case Study (FLICS) Club.” Journal of Leadership Studies 10, no. 4: 75–89. 10.1002/jls.21498.

[yd20658-bib-0031] Marturano, A. , J. T. Wren , and M. Harvey . 2013. “Editorial.” Leadership and the Humanities 1, no. 1: 1–5. 10.4337/lath.2013.01.00.

[yd20658-bib-0032] Mavin, S. 2009. “Gender Stereotypes And Assumptions: Popular Culture Constructions of Women Leaders.” https://ufhrd.co.uk/wp‐content/uploads/2009/07/6‐18‐refereed‐paper.pdf.

[yd20658-bib-0033] Owen, J. E. 2023. “Identity Development and Leadership: Emerging Intersections.” New Directions for Student Leadership 2023, no. 179: 33–45. 10.1002/yd.20556. 36945906

[yd20658-bib-0034] Roberts, D. C. 2007. Deeper Learning in Leadership: Helping College Students Find the Potential Within. Jossey‐Bass.

[yd20658-bib-0035] Scott, M. , and P. P. Weeks . 2016. “Using Film to Teach Authentic Leadership.” Journal of Leadership Education 15, no. 1: 140–149. 10.12806/v15/i1/a5.

[yd20658-bib-0036] Sinclair, A. 2014. “A Feminist Case for Leadership.” In Diversity in Leadership: Australian Women Past and Present, edited by J. Damousi , K. Rubenstein , and M. Tomsic , 17–35. Australian National University ePress. 10.22459/dl.11.2014.01.

[yd20658-bib-0037] Treisman, R. 2024. “Team USA Has Women to Thank for More Than Half of Its Olympic Medals.” Houston Public Media. https://www.houstonpublicmedia.org/articles/news/sports/2024/08/12/496314/team‐usa‐has‐women‐to‐thank‐for‐more‐than‐half‐of‐its‐olympic‐medals/.

[yd20658-bib-0038] Tutu, D. 1999. No Future Without Forgiveness. Doubleday.

[yd20658-bib-0039] Wilson, S. 2008. Research Is Ceremony: Indigenous Research Methods. Fernwood Publishing.

[yd20658-bib-0040] Zhou, X. 2024. “Breakthrough and Beyond: An Analysis of Feminism in Barbie.” In Proceedings of the 2nd International Conference on Social Psychology and Humanity Studies , 184–190. 10.54254/2753-7048/40/20240747.

